# Mr. Simmons, on Mr. White's Treatment of Sphacelus

**Published:** 1799-08

**Authors:** W. Simmons

**Affiliations:** Manchester


*To the Editors of the Medical and Phyſical Journal.*

GENTLEMEN,

IN the fifth Number of your Journal for July, p. 509, I obſerve that you notice muſk, and ſal cornu cervi volatilis, in equal proportions, as a new and efficacious remedy in ſphacelus, particularly in that ſpecies of it "which is accompanied with convulſive ſymptoms, and has ariſen from local external injury;" and you refer to a treatiſe on that ſubject, ſuppoſed to have been written by a Mr. C. WHITE, of York.

Permit

Permit me to acquaint you, that you have been misinformed relative to the author of the treatise alluded to; Mr. C. WHITE, of Manchester, having first promulgated to the world an account of that remedy in the year 1790, in a pamphlet entitled "*Observations on Gangrenes and Mortifications, accompanied with, or occasioned by, convulsive spasms, or arising from local injury, producing irritation.*"

Mr. White, however, was not the discoverer of the efficacy of musk and volatile salt in such cases, for the merit of the discovery is due to the late Dr. DARBEY, who casually hit upon it, in the treatment of a patient belonging to one of the other surgeons of the establishment, while he resided at the Manchester Infirmary, in the capacity of apothecary and house-surgeon.

In the same year in which Mr. White published his pamphlet, and very soon after its publication, Dr. Darbey graduated at Glasgow, and he made choice of this subject for his thesis, the title of which is "*Dissertatio Medica quædum de moschi et salis alk. volat, usu in febre nervosa et gangrena proponens.*"

Since that time, musk and volatile alkali combined, have been a good deal used in gangrene and sphacelus, by some practitioners resident in this place, and I suppose with success, as the practice is still continued; but as I have found the bark to answer very generally in gangrene and mortification, I have had no experience of it myself.

I have been induced to send you this communication, in consequence of your remark, that "the effects of this medicine are stated to be such as deserve the greatest attention of practitioners; but we do not find that it has been generally employed, unless by foreign practitioners, who speak of it in the highest terms of commendation;" and also to do justice to the memory the real author of the discovery, the history of which I have often heard him relate.

